# Germline variants predictive of tumor mutational burden and immune checkpoint inhibitor efficacy

**DOI:** 10.1016/j.isci.2021.102248

**Published:** 2021-03-04

**Authors:** Ajay Chatrath, Aakrosh Ratan, Anindya Dutta

**Affiliations:** 1 Department of Biochemistry and Molecular Genetics, University of Virginia, School of Medicine, 1240 Pinn Hall, Charlottesville, VA 22908, USA; 2 Center for Public Health Genomics, University of Virginia, Charlottesville, VA 22908, USA

**Keywords:** Genetics, Genomics, Cancer

## Abstract

High tumor mutational burden (TMB) is associated with response to checkpoint blockade in several cancers. We identify pathogenic germline variants associated with increased TMB (GVITMB). GVITMB were found in 7 genes using a pan-cancer approach (*APC*, *FANCL*, *SLC25A13*, *ERCC3*, *MSH6*, *PMS2,* and *TP53*) and 38 gene sets (e.g., those involved in DNA repair and programmed cell death). GVITMB were also associated with mutational signatures related to the dysfunction of the gene carrying the variant, somatic mutations that further affect the gene or pathway with the variant, or transcriptomic changes concordant with the expected effect of the variant. In a validation cohort of 140 patients with cutaneous melanoma, we found that patients with GVITMB had prolonged progression-free survival (p = 0.0349, hazard ratio = 0.688) and responded favorably (p = 0.0341, odds = 1.842) when treated with immune checkpoint inhibitors. Our results suggest that germline variants can influence the molecular phenotypes of tumors and predict the response to immune checkpoint inhibitors.

## Highlights

• GVITMB were found in 7 genes and 38 gene sets
• GVITMB influence the somatic mutation and gene expression profiles of tumors
• GVITMB predict immune checkpoint inhibitory efficacy in SKCM

## Graphical abstract

**Figure:**
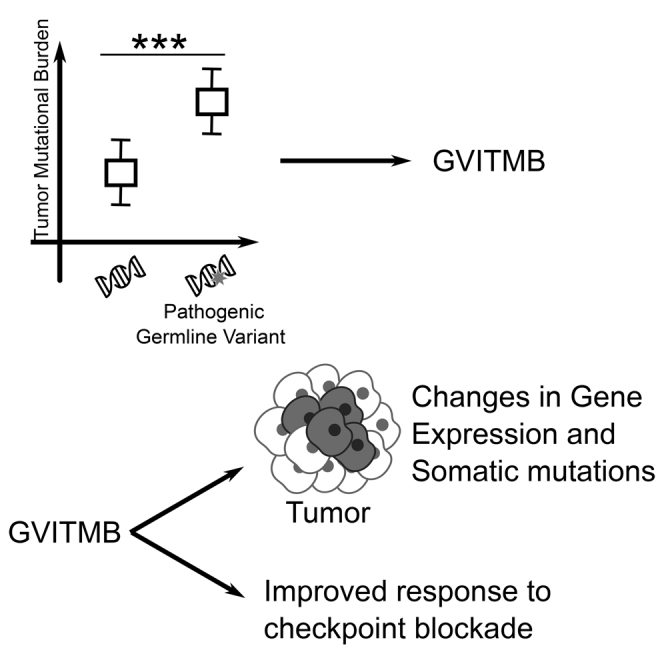

## Introduction

The explosion of massively parallel sequencing data has helped to identify rare germline variants that cause or contribute to disease (Sanderson et al., 2019; Vaske et al., 2019). In oncology, it is well-established that patients with germline variants in genes mutated in certain genetic syndromes, such as Lynch syndrome, Li-Fraumeni syndrome, von Hippel-Lindau syndrome, and Fanconi anemia, are at much higher risk of acquiring cancer (Ellrott et al., 2018; Kamps et al., 2017). Although individuals with these pathogenic germline variants are generally screened more aggressively, clinical management of patients with these germline variants is not always differentiated from the management of patients without these pathogenic variants (Ballinger et al., 2017; Lindor et al., 2006; Maher et al., 1990). This has begun to change. For example, patients with Lynch syndrome have pathogenic germline variants in mismatch repair genes, such as *MSH2*, *MSH6*, *PMS2*, and *MLH1,* and their tumors exhibit higher levels of microsatellite instability. As a consequence, patients with Lynch syndrome are more likely to respond to immune checkpoint inhibitors such as pembrolizumab (Snyder et al., 2014; Van Allen et al., 2015) (Le et al., 2017).

We have reported that germline variants affect tumor progression across a large spectrum of cancers through the analysis of common germline variants with a minor allele frequency greater than 5% in the general population (Chatrath et al., 2019, 2020). In this study, we analyze rare pathogenic germline variants to identify germline variants associated with increased tumor mutational burden (GVITMB) to test whether these germline variants increase the likelihood of a patient responding to immune checkpoint inhibitors (Keenan et al., 2019; Liu et al., 2019; Miao et al., 2018). After identifying the set of pathogenic germline variants predictive of tumor mutational burden (TMB), we demonstrate that they predict responsiveness to immune checkpoint inhibitors in a cohort of 140 patients with skin cutaneous melanoma.

## Results

### Germline variants can be analyzed using pan-cancer or gene set-level approaches

Huang et al. had previously described 435 rare pathogenic germline variants that were found in the patients in The Cancer Genome Atlas (TCGA) (Huang et al., 2018). Briefly, all somatic variants were scored based on the American College of Medical Genetics and Genomics and the Association for Molecular Pathology (ACMG-AMP) guidelines developed for rare variants in cancer and variants known to be pathogenic in ClinVar and curated databases were labeled as pathogenic. The majority of these pathogenic germline variants were predicted to functionally perturb known tumor suppressor genes or oncogenes. Before identifying which pathogenic germline variants contribute to elevated TMB, we first evaluated whether we were able to identify GVITMB based in individual genes in individual cancers. We set a modest threshold requiring at least five patients in the cancer cohort to have a pathogenic germline variant in a given gene.

**Figure 1:**
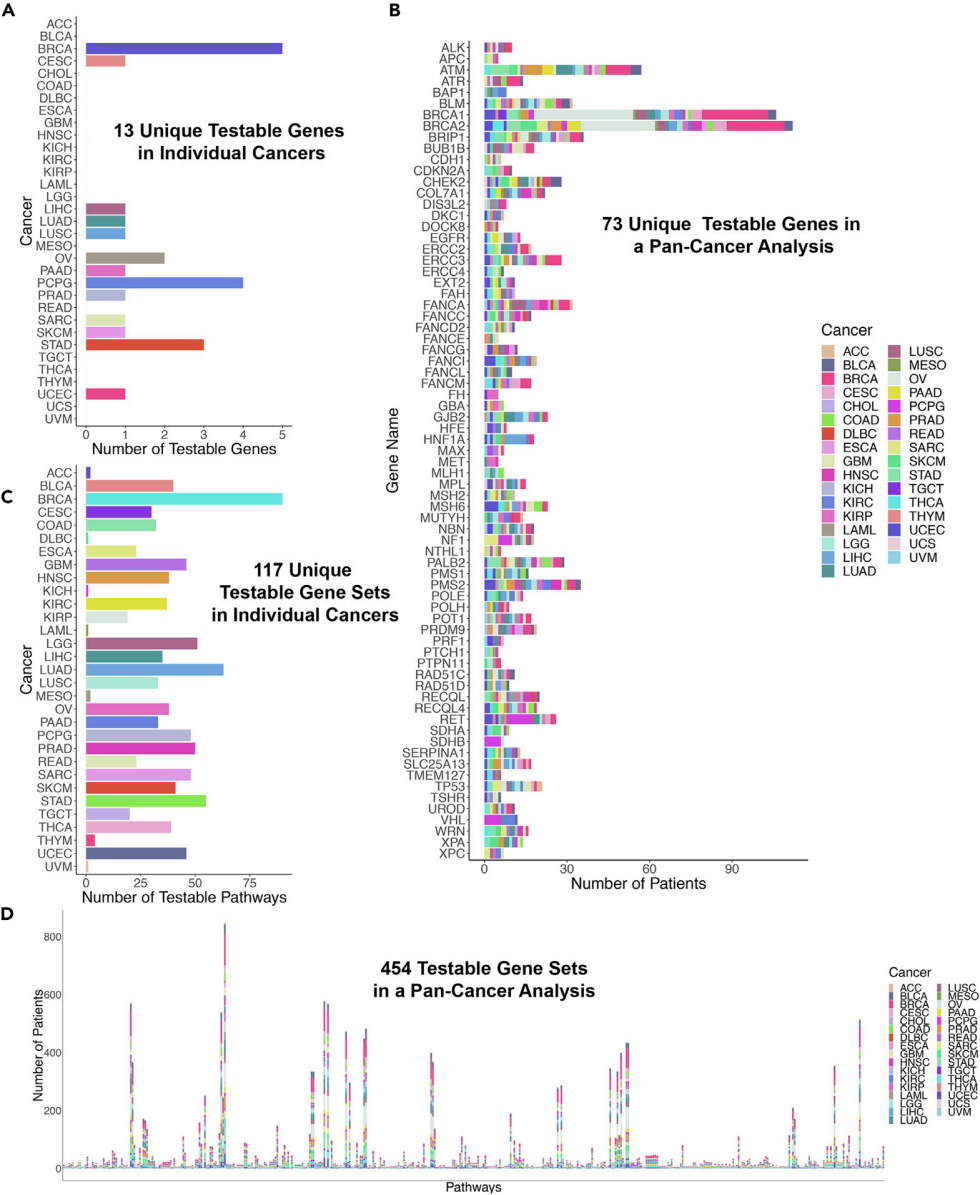
An overview of the number of genes or gene sets that could be tested with the threshold that the pathogenic germline variants must be present in five or more patients (A) Number of testable genes in individual cancer types. This analysis was not performed due to the small number of testable genes. (B) Number of patients with pathogenic variants in the indicated genes when patients with all cancers were pooled together. The stacked bars show the cancer types color coded as in the key. These patients were analyzed by Approach 1. (C) Number of testable gene sets in each of the individual cancer types, analyzed by Approach 2. (D) Number of patients carrying germline variants in the testable gene sets, analyzed by Approach 3. The stacked bars show the cancer types with pathogenic germline variants in a given gene set color coded as in the key.

**Figure 2:**
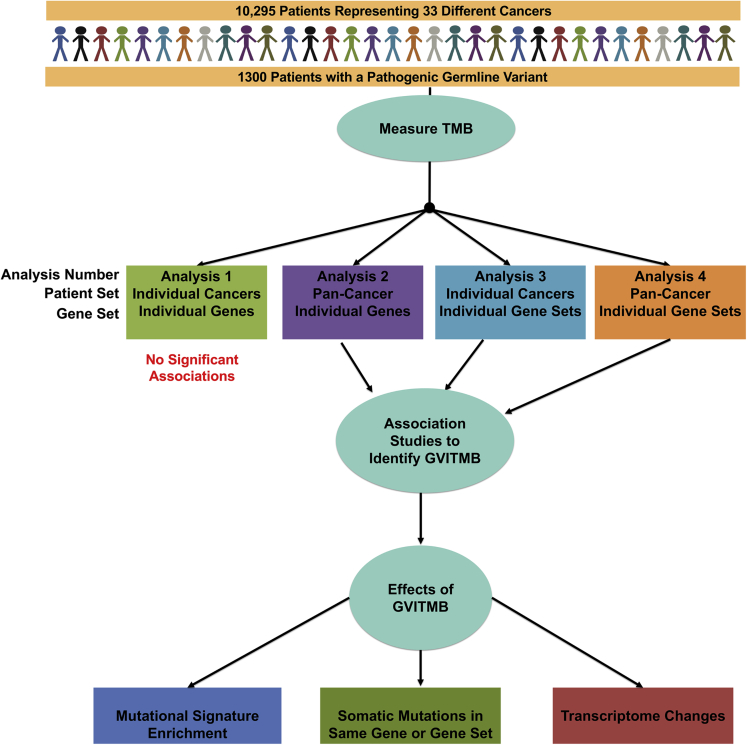
A summary of the overall approach employed in this study

We utilized four approaches to identify germline variants associated with increased TMB. (1) We tested individual genes for association with TMB in individual cancers, testing a total of 13 unique genes (Figure 1A). (2) We pooled all the patients in TCGA together, and by doing so we were now able to test 73 total genes for the presence of GVITMB (Figure 1B). (3) We grouped the pathogenic germline variants by gene set to identify gene sets carrying GVITMB in individual cancers (Figure 1C). (4) Finally, we repeated the analysis in (3) but after grouping all cancers together (Figure 1D). Our overall methodology is summarized in Figure 2.

### Calculation of tumor mutational burden

**Figure 3:**
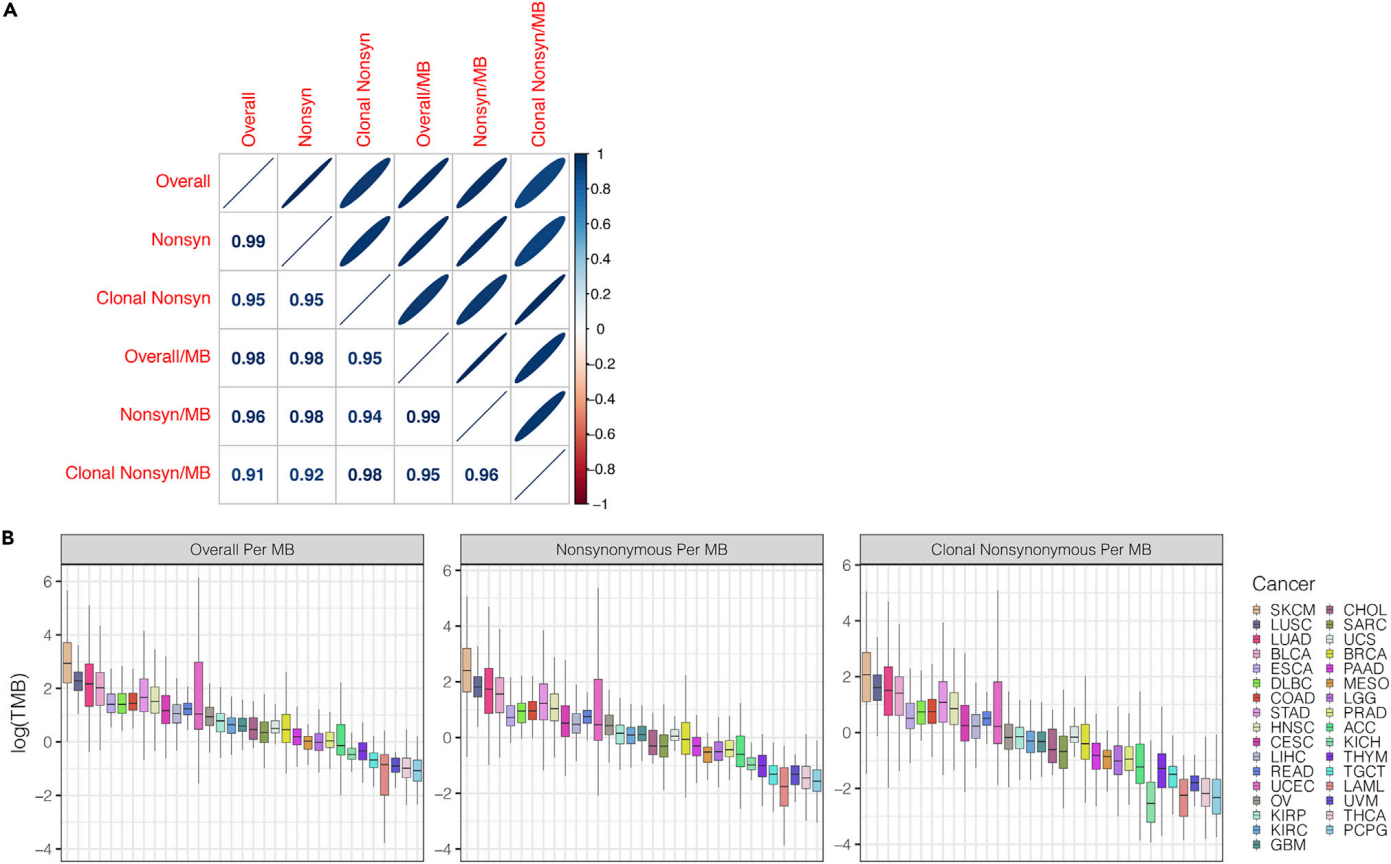
Calculated tumor mutational burden across cancers (A) All six metrics of tumor mutational burden are highly correlated with each other. (B) Overall TMB per megabase (MB), nonsynonymous TMB per MB, and clonal nonsynonymous TMB per MB across cancers.

Overall TMB, nonsynonymous TMB, and clonal nonsynonymous TMB have previously been reported to be associated with favorable response in patients treated with immune checkpoint inhibitors (Keenan et al., 2019; Miao et al., 2018). We, therefore, calculated these three metrics of TMB for each patient in TCGA and normalized them to per megabase (MB) based on the total number of sites in each patient wherein we were sufficiently powered to call a somatic mutation. This normalization accounted for the coverage at each site in the exome and the purity and ploidy of each tumor. All metrics of TMB were highly correlated to each other (Spearman’s rho >0.90 for all pairs, Figure 3A), and we present the normalized distribution of TMB by cancer in Figure 3B. We used clonal nonsynonymous TMB per MB as our dependent variable for this study as it has been shown to have a better association with immune checkpoint inhibitor responsiveness (Keenan et al., 2019; Miao et al., 2018).

### Pan-cancer identification of individual genes associated with TMB

**Figure 4:**
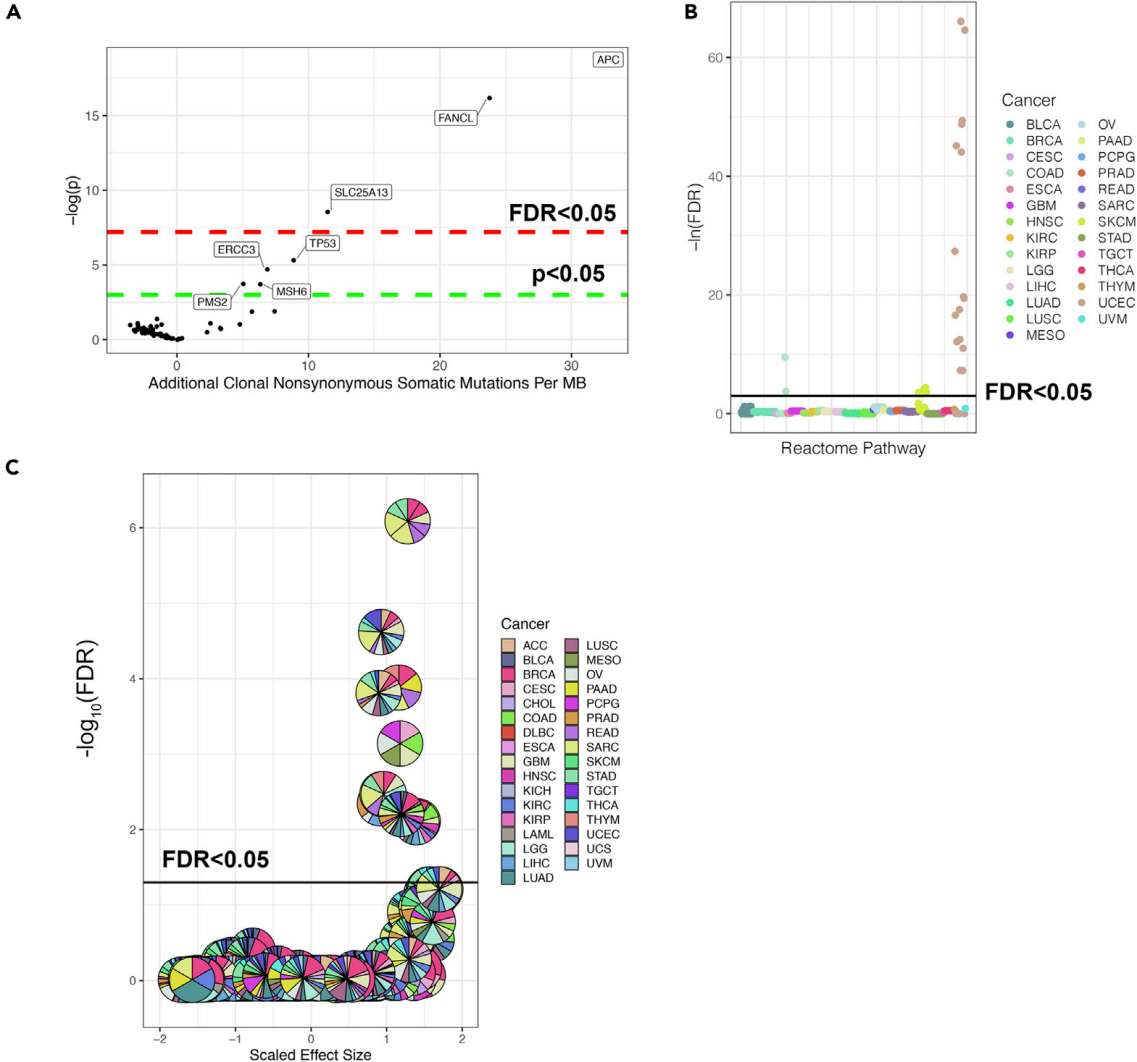
Manhattan plots summarizing the associations with clonal nonsynonymous tumor mutational burden per megabase (A–C) We identified associations with elevated tumor mutational burden in (A) genes perturbed by pathogenic germline variants using a pan-cancer approach, (B) gene sets perturbed by pathogenic germline variants in individual cancers, and (C) gene sets perturbed by pathogenic germline variants using a pan-cancer approach. For each gene set, the fraction of patients with a particular cancer carrying a pathogenic germline variant is indicated by the color code.

We identified seven genes that when perturbed by a pathogenic germline variant are associated with elevated TMB (Figure 4A, Table 1). Three of these genes (*APC*, *FANCL*, and *SLC25A13*) were determined to be significant after multiple hypothesis testing correction (adjusted p value <0.05). However, later in this study we also characterize the four genes (*ERCC3*, *MSH6*, *PMS2*, and *TP53*) that did not reach the significance threshold of an adjusted p value < 0.05 even though they crossed the raw p value threshold of <0.05 because they have well-known roles in DNA repair.

**Table 1:** A summary of the associations we found with elevated somatic mutation burden in individual genes using a pan-cancer approach

Gene	Number of patients with a PGV	Additional clonal nonsynonymous mutations per MB	p value	Adjusted p value
APC	5	32.51	7.910E-09	5.300E-07
FANCL	8	23.77	9.500E-08	3.180E-06
SLC25A13	17	11.46	1.938E-04	4.329E-03
TP53	16	8.87	4.897E-03	8.202E-02
ERCC3	23	6.87	9.044E-03	1.212E-01
MSH6	20	6.34	2.453E-02	2.348E-01
PMS2	32	5.05	2.367E-02	2.348E-01

PGV, Pathogenic Germline Variants.

### Identification of gene sets carrying GVITMB in individual cancers

We identified significant associations of pathogenic germline variants in gene sets and TMB in Colon Adenocarcinoma (COAD), Skin Cutaneous Melanoma (SKCM), and Uterine Corpus Endometrial Carcinoma (UCEC) (Figure 4B, Table 2, list of perturbed genes in Table S1). Although each of the identified gene sets consisted of different and unique gene sets, the genes that empirically contributed to these gene sets sometimes overlapped in this analysis. We have therefore grouped gene sets for which the contributing genes entirely overlapped in this particular analysis. In total, we identified 29 associations (2 in COAD, 11 in SKCM, and 16 in UCEC). The significantly associated gene sets were primarily related to DNA damage and repair and cell cycle control.

**Table 2:** A summary of the associations we found with elevated somatic mutation burden in individual gene sets in individual cancers

Gene set	Cancer	Patients with PGV	Additional clonal nonsynonymous mutations per MB	p value	Adjusted p value
TP53 regulates transcription of DNA repair genes	UCEC	14	38.78	8.712E-31	2.004E-29
DNA repair	UCEC	31	25.71	7.358E-30	8.462E-29
Transcriptional regulation by TP53	UCEC	20	27.86	4.704E-23	3.607E-22
Generic transcription pathway	UCEC	22	26.34	1.132E-22	6.508E-22
Disease	UCEC	12	34.18	5.317E-21	2.446E-20
Gene expression transcription	UCEC	24	23.87	1.828E-20	7.009E-20
Mismatch repair, diseases of mismatch repair (MMR)	UCEC	7	34.55	3.925E-13	1.290E-12
Sumoylation	UCEC	6	31.43	9.928E-10	2.854E-09
Deubiquitination	UCEC	6	31.15	1.396E-09	3.568E-09
Fanconi anemia pathway	UCEC	8	25.54	1.040E-08	2.393E-08
DNA double-strand break response	UCEC	7	26.42	2.932E-08	6.130E-08
G2 M checkpoints, G2 M DNA damage checkpoint, regulation of TP53 activity, regulation of TP53 activity through phosphorylation	UCEC	9	20.00	1.958E-06	3.753E-06
Post-translational protein modification	UCEC	9	19.61	3.065E-06	5.423E-06
Cell cycle checkpoints	UCEC	10	17.62	9.955E-06	1.635E-05
Disease	COAD	8	20.53	4.064E-06	7.315E-05
DNA double-strand break repair	UCEC	15	11.48	4.291E-04	6.580E-04
Cell cycle	UCEC	15	11.37	4.835E-04	6.950E-04
DNA repair	SKCM	30	7.89	6.214E-04	1.243E-02
Disease	SKCM	6	16.17	1.680E-03	1.680E-02
Cell cycle	SKCM	20	8.43	2.805E-03	1.870E-02
Generic transcription pathway, gene expression transcription	COAD	15	9.86	2.467E-03	2.220E-02
Cell cycle checkpoints	SKCM	12	9.57	8.553E-03	2.851E-02
Regulation of TP53 activity	SKCM	11	10.38	6.340E-03	2.851E-02
DNA double-strand break repair	SKCM	19	7.72	7.643E-03	2.851E-02
Homology-directed repair (HDR) through homologous recombination (HRR)	SKCM	17	7.85	1.027E-02	2.903E-02
Resolution of D-loop structures, resolution of D-loop structures through synthesis-dependent strand annealing (SDSA), homologous DNA pairing, and strand exchange	SKCM	16	7.96	1.161E-02	2.903E-02
Generic transcription pathway, gene expression transcription	SKCM	18	6.90	2.029E-02	3.568E-02
G2 M checkpoints, G2 M DNA damage checkpoint, regulation of TP53 activity through phosphorylation	SKCM	10	9.52	1.696E-02	3.568E-02
Transcriptional regulation by TP53	SKCM	16	7.44	1.827E-02	3.568E-02

### Pan-cancer identification of gene sets carrying GVITMB

Last, we identified pathogenic germline variants associated with TMB using a pan-cancer approach in which the pathogenic germline variants were grouped by gene set (Figure 4C, Table 3, list of perturbed genes in Table S2). In total, we identified 12 significant associations. Several of the gene sets were related to *Wnt* signaling, and the pathogenic germline variants in *APC* greatly contributed to these associations, as described in our analysis of individual genes. One association was driven entirely by *SLC25A13* and had also been described in our previous analysis of individual genes. The other associations were related to apoptosis, cell cycle control, and DNA damage repair.

**Table 3:** A summary of the associations we found with elevated somatic mutation burden in individual gene sets using a pan-cancer approach

Gene set	Number of patients with PGV	Additional clonal nonsynonymous mutations per MB	p value	Adjusted p value
Degradation of β-catenin by the destruction complex	5	32.51	7.907E-09	8.223E-07
β-catenin phosphorylation cascade, disassembly of the destruction complex and recruitment of axin to the membrane, signaling by WNT in cancer, phosphorylation site mutants of CTNNB1 are not targeted to the proteasome by the destruction complex	5	32.51	7.907E-09	8.223E-07
Ovarian tumor domain proteases	22	13.70	3.466E-07	2.403E-05
Deactivation of the β-catenin transactivating complex	7	22.42	2.517E-06	1.309E-04
Programmed cell death	28	11.03	3.729E-06	1.551E-04
Regulation of kit signalling	5	23.99	2.086E-05	7.231E-04
Apoptotic cleavage of cellular proteins, apoptotic execution phase	11	14.55	1.299E-04	3.378E-03
Signaling by WNT, TCF-dependent signaling in response to WNT	10	15.31	1.241E-04	3.378E-03
Mitochondrial protein import, gluconeogenesis, glucose metabolism, aspartate and asparagine metabolism, protein localization	17	11.46	1.938E-04	4.480E-03
Disease	211	3.17	2.993E-04	6.226E-03
Mismatch repair	63	5.63	4.116E-04	7.782E-03
Diseases of mismatch repair (MMR)	62	5.62	4.615E-04	7.999E-03

### GVITMB influence somatic events

We next sought to characterize the somatic events associated with GVITMB. Several studies have suggested that germline variants influence somatic events (Carter et al., 2017; Chatrath et al., 2019, 2020; Chirita-Emandi et al., 2020). We found that patients with GVITMB in mismatch repair genes exhibited enrichment of mutational signatures associated with mismatch repair gene dysfunction, suggesting exome-wide evidence of the dysfunction of these genes (Table 4).

**Table 4:** Mutational signature results concordant with the expected effects of the pathogenic germline variants

Gene or gene set	Cancer	Mutational signature	Fold enrichment	p value
MSH6	Pan-cancer	44	3.83	3.11E-03
Mismatch repair	UCEC	20	2.16	2.90E-02
Mismatch repair	Pan-cancer	20	2.16	2.13-03
Mismatch repair	Pan-cancer	26	1.58	3.48E-02
Mismatch repair	Pan-cancer	44	2.89	8.38E-06

We next tested whether the genes and gene sets perturbed by GVITMB were associated with somatic mutations in these same genes or gene sets. We controlled for TMB in all analyses to account for the general increase in somatic mutations in tumors with the GVITMB, along with controlling for tumor type and demographic factors. Patients with GVITMB in the mismatch repair gene *PMS2* were much more likely to exhibit somatic mutations in *PMS2* than patients without the GVITMB in *PMS2* (beta = 3.05, p value = 5.86E-5, adjusted p value=4.1E-4). We found that GVITMB in *ERCC3* or *TP53* were associated with an increased incidence of somatic mutations in gene sets that include *ERCC3* or *TP53*, respectively (Table S3). In addition, patients with SKCM with GVITMB in the disease gene set (a compilation of genes associated with human diseases) were more likely to acquire somatic mutations in other genes of the same gene set (beta=20.2, p value = 4.12E-6, adjusted p value=1.73E-4).

Finally, we tested for up- or downregulation of gene expression consistent with the expected effects of the GVITMB. We found that patients with GVITMB in genes regulating the G2-M checkpoint in UCEC exhibited upregulation of E2F target genes, suggesting upregulation of cell cycle activity (p value = 0.013).

### GVITMB predict immune checkpoint inhibitory efficacy in SKCM

**Figure 5:**
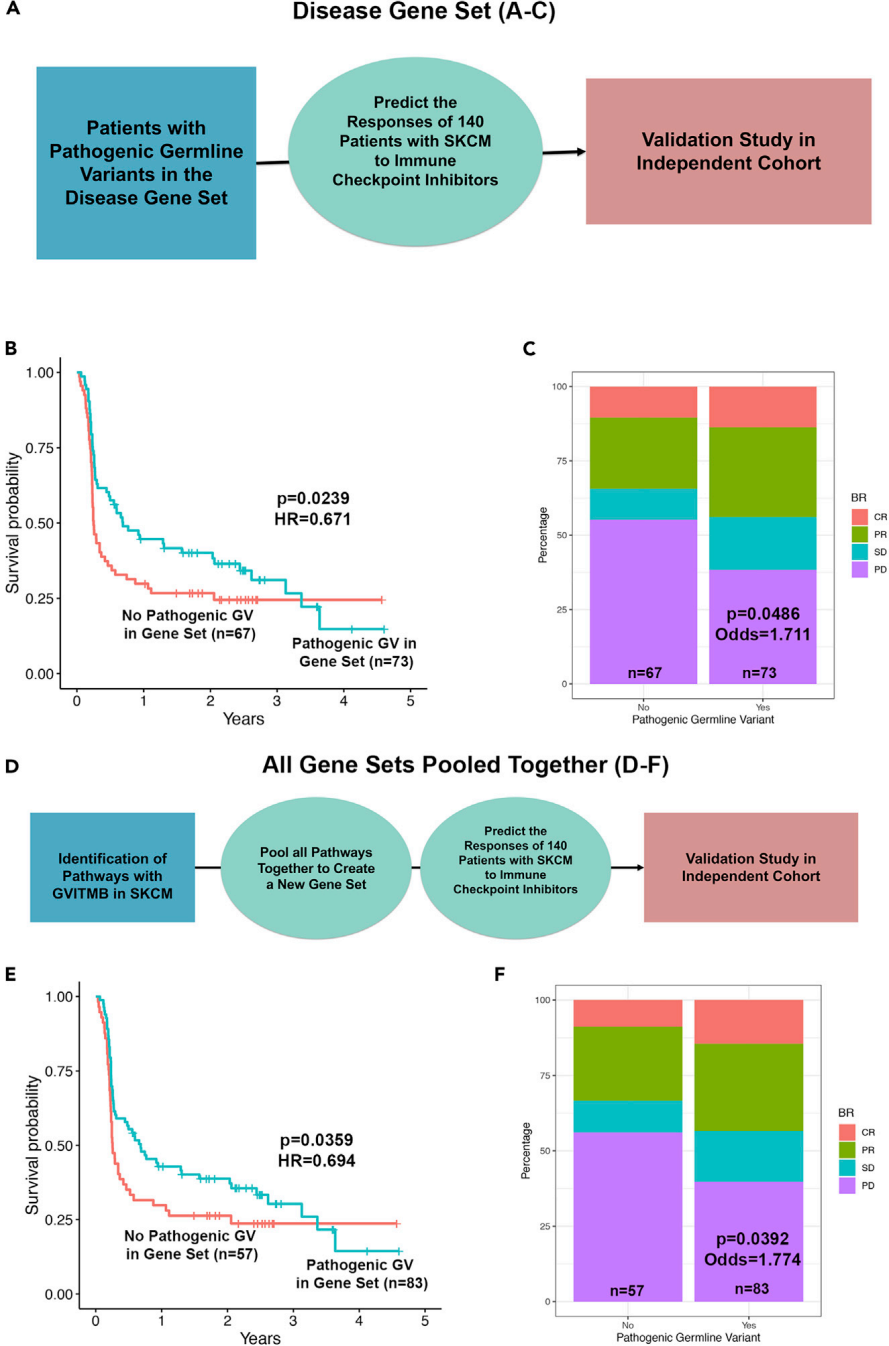
Pathogenic germline variants predict immune checkpoint inhibitor efficacy in an independent cohort of 140 patients with skin cutaneous melanoma treated with immune checkpoint inhibitors (A–C) Patients with pathogenic germline variants in the (A) disease gene set exhibit (B) prolonged progression-free survival and (C) are more likely to respond to immune checkpoint inhibitors. (D–F) We (D) pooled all gene sets with GVITMB in SKCM together and found that patients with germline variants in these gene sets exhibited (E) prolonged progression-free survival and are (F) more likely to respond to immune checkpoint inhibitors. Abbreviations: PD, progressive disease; SD, stable disease; PR, partial response; CR, complete response.

To test whether patients with SKCM with pathogenic germline variants in the gene sets that we had found to be associated with TMB in the TCGA dataset (Table 2) responded better to immune checkpoint inhibitors, we analyzed sequencing data from 140 patients with SKCM treated with either nivolumab or pembrolizumab (Liu et al., 2019). Given the relatively small sample size, we were not sufficiently powered to test individual gene sets for association with outcome. Of all the gene sets that contained GVITMB in SKCM (Table 2), only the disease gene set was sufficiently powered to detect an association with progression-free survival. Patients with pathogenic germline variants in the disease gene set exhibited prolonged progression-free survival (p = 0.0245, hazard ratio [HR] = 0.662) (Figures 5A and 5B) and were more likely to show a response to immune checkpoint inhibitors based on Response evaluation criteria in solid tumors (RECIST) criteria (p = 0.0393, odds = 1.781, ordering of categories was progressive disease, stable disease, partial response, and then complete response) (Figure 5C). Although patients with pathogenic germline variants had a higher median number of overall mutations, nonsynonymous mutations, and clonal nonsynonymous mutations, this difference was not statistically significant (Table S4, top three rows).

We were better powered to detect such an association by pooling all pathogenic germline variants found in the genes of the gene sets that we found to be associated with elevated TMB in SKCM the TCGA dataset (Figures 5D and Table 2). When tested, we found that patients with pathogenic germline variants in these genes exhibited favorable outcome and were less likely to progress (Figure 5E, p = 0.0349, HR = 0.688). Similarly, patients with pathogenic germline variants in these genes were more likely to exhibit a response to immune checkpoint inhibitors based on RECIST criteria (Figure 5F, p = 0.0341, odds = 1.842). Turning to TMB, we found that the median number of total mutations, nonsynonymous mutations, and clonal nonsynonymous mutations was greater in patients with pathogenic germline variants in genes in our gene set than patients without these pathogenic germline variants, although these differences were also not statistically significant (Table S4, lower three rows). Thus the GVITMB have a more significant effect on responsiveness than can be expected from the differences in TMB alone.

## Discussion

The widespread collection of sequencing data has enabled detailed study of rare genetic syndromes (Kamps et al., 2017; Sylvester et al., 2018). Although patients with pathogenic germline variants are often screened more aggressively for cancer, clinical guidelines for these patients have only changed in a few circumstances (Le et al., 2017; Lindor et al., 2006). We had previously identified common germline variants associated with differences in patient outcome across a multitude of cancers, suggesting that germline variation contributes not only to cancer risk but also to tumor progression (Chatrath et al., 2019, 2020). In this study, we have identified pathogenic germline variants associated with TMB. Some of these associations were expected and confirmed existing hypotheses (e.g., mutations in known DNA repair genes such as *MSH6* and *PMS2*), whereas other associations (e.g., mutations in *SLC25A16*) are more surprising and can motivate future hypotheses. We identified molecular fingerprints of the effects of some of the pathogenic germline variants by analyzing RNA sequencing data and somatic mutation profiles. Our findings suggest that these pathogenic germline variants remain relevant after a patient has been diagnosed with cancer and may contribute to the molecular differences in tumors collected from patients with and without pathogenic germline variants.

After identifying the set of pathogenic germline variants associated with TMB in skin cutaneous melanoma, we showed that patients with these pathogenic germline variants exhibit prolonged progression-free survival and increased responsiveness to immune checkpoint inhibitors. Given the relatively small size of the validation cohort, our validation study had limited resolution because we were not adequately powered to test individual genes or gene sets. As the total amount of sequencing data from patients treated with immune checkpoint inhibitors continues to increase, our ability to identify individual genes and gene sets predictive of responsiveness will improve. In this study, we identify pathogenic germline variants associated with TMB as a proxy for immune checkpoint inhibitory efficacy, although determining the extent to which TMB is predictive of immune checkpoint inhibitor efficacy across all cancers is still an active area of research (Wood et al., 2020).

Tumors from patients with pathogenic germline variants in the mismatch repair genes *MSH6* and *PMS2* and in the mismatch repair pathway exhibit elevated TMB. We found enrichment in the contribution to these patients’ somatic mutation profiles from COSMIC signatures related to mismatch repair. Germline mismatch repair deficiency has previously been associated with microsatellite instability and increased responsiveness to immune checkpoint inhibitors, and so these findings served as an important positive control in our study (Le et al., 2017).

Tumors with pathogenic germline variants in the nucleotide excision repair gene *ERCC3* were associated with elevated TMB in our study. Although a previous study showed that somatic mutations in the nucleotide base excision repair gene *ERCC2* likely contributes to increased TMB, no previous study has demonstrated an association between nucleotide excision repair gene perturbation and immune checkpoint inhibitor efficacy (Van Allen et al., 2014). We did not find a significant association between nucleotide excision repair pathway perturbation by pathogenic germline variants and TMB at the pathway level, suggesting that the contribution to TMB may be limited to select genes in the pathway.

We found patients with pathogenic germline variants in *APC*, which binds to beta-catenin and leads to its degradation, and genes involved with beta-catenin degradation to be associated with elevated somatic mutation burden. Aberrations to the *Wnt* signaling pathway are linked to the formation of many cancers (Anastas and Moon, 2013). Spranger et al. showed that non-T cell inflamed tumors exhibited high β-catenin signaling activity and reduced response to immune checkpoint blockade (Spranger et al., 2015). Further work is necessary to predict whether pathogenic germline variants in *APC* and genes involved with β-catenin degradation will be associated with increased or decreased response to immunotherapy, as the elevated TMB would be expected to increase efficacy, whereas the elevated β-catenin signaling would be expected to decrease efficacy.

Tumors from patients with pathogenic germline variants in *SLC25A13* exhibited elevated somatic mutation burden. This gene codes for a mitochondrial aspartate/glutamate transporter. Pathogenic germline variants in this gene are associated with the urea cycle disorder type II citrullinemia and neonatal intrahepatic cholestasis (Song et al., 2013). Lee et al. have previously shown that tumors exhibiting urea cycle dysfunction generate nitrogen metabolites, resulting in DNA damage and ultimately better response to immune checkpoint blockade (Lee et al., 2018). Lee et al.’s analysis focused on somatic urea cycle dysfunction, whereas our work suggests that germline urea cycle dysfunction may also be a marker for improved immune checkpoint blockade response.

FANCL is the E3 ubiquitin ligase subunit within the FA core complex that enhances the efficiency of FANCD2 monoubiquitination. FANCD2 participates in DNA damage recognition and repair. As the pathogenic germline mutations in FANCL associated with TMB are predicted to be loss-of-function mutations, we hypothesize that they lower the efficacy of interstrand crosslink repair, affecting TMB.

High TMB has been associated with response to checkpoint blockade in several malignancies. However, the degree to which TMB changes over time, across anatomical sites, and with intervening treatment is still not clear. Studies have noted that tumor sampling from different anatomical sites may be associated with greater discrepancies in TMB calculations (Smithy et al., 2019). Efforts are ongoing to standardize TMB evaluation, which is needed to ensure reliability, reproducibility, and clinical utility (Galuppini et al., 2019). Compared with TMB, germline variants are relatively simpler to detect, annotate, score, and classify (Huang et al., 2018). Furthermore, they do not change during the course of the disease. It remains to be evaluated if they have additional value as a biomarker beyond that is provided by TMB, but our analyses suggest that they should be viewed as a biomarker candidate that can provide a robust and reproducible signal.

Overall, the results of our analysis suggest that understanding the germline contribution to somatic events could inform clinical therapy decisions (Carter et al., 2017; Menden et al., 2018). In this study, we have shown that pathogenic germline variants inform TMB and that these sets of pathogenic germline variants can be used to predict immune checkpoint inhibitor efficacy in patients with skin cutaneous melanoma. Future studies of germline variants in cancer will likely continue to illuminate areas in which clinical management can be further personalized based on an understanding of a patient's germline variants.

### Limitations of the study

In this study, we used the TCGA data to identify pathogenic germline variants that are associated with increased tumor mutation burden (GVITMB). More than 80% of the patients in TCGA are of European ancestry, so it remains to be seen whether these associations will be replicated in a more diverse cohort. For the association analysis, we collapse the pathogenic variants in genes and gene set with the assumption that all pathogenic germline variants contribute toward increased TMB. It is likely that using adaptive burden association tests could increase our power to determine the associations, but that would come at the expense of interpretability. Using a second SKCM dataset, we were able to show that GVITMB have prognostic value, but it still needs to be determined whether GVITMB offer additional prognostic value beyond TMB. However, GVITMB do offer some advantages, as we highlight in the discussion, and should be considered as possible biomarker candidates in future studies.

### Resource availability

#### Lead contact

Further information and questions should be directed to and will be fulfilled by the lead contact, Anindya Dutta (ad8q@virginia.edu).

#### Materials availability

This study did not generate new unique reagents.

#### Data and code availability

All scripts used for analyses are available at https://github.com/achatrath/GermlineSomaticMutationBurden.

## Methods

All methods can be found in the accompanying Transparent Methods supplemental file.

## Supplemental information

Document S1. Transparent methods

Table S1. Pathways in table 2 expanded to include the genes perturbed by pathogenic germline variants in these pathways, related to table 2

Table S2. Pathways in table 3 expanded to include the genes perturbed by pathogenic germline variants in these pathways along with the number of patients with each cancer type, related to table 3

Table S3. Gene sets with an increased incidence of damaging somatic mutations in patients with ERCC3 or TP53 pathogenic germline variants, related to figure 4

Table S4 Number of somatic mutations in patients with and without pathogenic germline variants in the test gene set in a cohort of 140 patients diagnosed with SKCM treated with immune checkpoint inhibitors, related to figure 5
