# Supplementary material for: Germline variants predictive of tumor mutational burden and immune checkpoint inhibitor efficacy
Source: iScience. 2021 Mar 4;24(3):102248. doi: 10.1016/j.isci.2021.102248 (PMC7988326; doi:10.1016/j.isci.2021.102248)
Supplement: Document S1. Transparent methods [file mmc1.pdf]

iScience, Volume *24*

Supplemental information

Germline variants predictive of tumor mutational burden and immune checkpoint inhibitor efficacy

Ajay Chatrath, Aakrosh Ratan, and Anindya Dutta

Transparent Methods

Patient Data Availability

We downloaded the set of rare, pathogenic germline variants found in the patients in The Cancer Genome Atlas (TCGA) previously published by Huang et al. and the set of somatic mutations in these patients generated by Ellrott et al. (Ellrott et al., 2018; Huang et al., 2018). Clinical data for the TCGA patients were accessed from the TCGA pan-cancer clinical data resource (Liu et al., 2018). We used the calculated race of each patient from The Cancer Genome Ancestry Atlas to effectively control for genetic ancestry throughout our analyses (Yuan et al., 2018).

Calculating Tumor Mutational Burden (TMB)

We counted the number of somatic mutations called by Ellrott et al. in each patient in The Cancer Genome Atlas (Ellrott et al., 2018). This count depends on the number of sites in the exome where we are adequately powered to call a somatic mutation. We first determined the sequencing depth at each position in the exome for each patient using SAMtools (Lau et al., 2017; Li et al., 2009). We then estimated the power to detect a somatic mutation at each position in the exome for each patient using the R package PureCN utilizing the purity and ploidy previously reported by The Cancer Genome Atlas Pan-Cancer Atlas initiative (https://gdc.cancer.gov/about-data/publications/pancanatlas) (Riester et al., 2016). The somatic mutations had previously been categorized as synonymous or nonsynonymous by Ellrott et. al. (Ellrott et al., 2018). We classified the somatic mutations as clonal or subclonal by calculating the probability of observing the number of reads supporting the somatic mutation in the tumor based on a binomial distribution, given the total number of reads covering that

position. We assumed that the probability of a read supporting a clonal somatic mutation was equal to $\frac{1}{Ploidy*Tumor\ Purity}$. If the probability was less than 5% ($p<0.05$), we classified the somatic mutation as subclonal (Carter et al., 2012).

We calculated six metrics of tumor mutational burden. The first three were overall tumor mutational burden, nonsynonymous tumor mutational burden, and clonal nonsynonymous tumor mutational burden. These three metrics were normalized by the number of sites for which there was 80% or greater power to detect a somatic mutation. This enabled us to calculate overall tumor mutational burden per megabase, nonsynonymous tumor mutational burden per megabase, and clonal nonsynonymous tumor mutational burden per megabase. We used clonal nonsynonymous tumor mutational burden per megabase as our dependent variable for this study as clonal nonsynonymous TMB has been shown to be more closely associated with response to immune checkpoint inhibitors (Keenan et al., 2019; Liu et al., 2019; Miao et al., 2018).

Identification of Genes with GVITMB

Across all of the TCGA patients, 132 unique genes contained at least one pathogenic germline variant. We limited our analysis only to genes with pathogenic germline variants in at least five different patients. Our results do not change substantially when we lower this threshold. However, setting the minimum threshold at five patients eliminates associations driven by a small number of patients, which could make them less compelling and more difficult to validate. We tested individual genes for association with clonal nonsynonymous tumor mutational burden per megabase, controlling for age, gender (if applicable), and calculated patient race. We tested a total of 13 unique genes.

We also looked for associations between individual genes and tumor mutational burden using a pan-cancer approach. We pooled all of the TCGA patients together and tested whether individual genes perturbed by pathogenic germline variants (presence or absence of a pathogenic germline variant) were associated with clonal nonsynonymous TMB per megabase using linear regression, controlling for tumor type, age, gender, and calculated patient race. We tested a total of 73 unique genes in this analysis. P-values were adjusted using the Benjamini-Hochberg procedure throughout this study.

Identification of Gene Sets with GVITMB

To study the association between pathogenic germline variants and tumor mutational burden in individual cancers, we grouped genes by gene sets. Gene sets perturbed by pathogenic germline variants in five or more patients were tested. Gene set annotation was downloaded from Reactome (Fabregat et al., 2018). We tested whether having a pathogenic germline variant in the gene set (presence or absence) was associated with clonal nonsynonymous TMB per megabase using linear regression, controlling for age, gender, and calculated patient race. We tested a total of 117 unique gene sets. Finally, we performed a pan-cancer analysis of gene sets associated with clonal nonsynonymous TMB per megabase using the same approach, controlling for tumor type, age, gender, and calculated patient race. We tested a total of 454 unique gene sets in this analysis. While each gene set included in these analyses is unique, some of the gene sets have overlapping sets of genes (**Table 2-3** and **Table S1-2**).

Gene Set Enrichment Analysis

We performed gene set enrichment analysis to test for upregulation or downregulation of RNAs in specific gene sets in patients with GVITMB. To do this, we downloaded the previously released RNA-sequencing quantification files for each patient generated by the TCGA research network (https://portal.gdc.cancer.gov/). We then excluded genes with a median expression level of <1 FPKM across the patient cohort being tested. The expression values of the remaining genes were then standardized to have a mean of 0 and a standard deviation of 1. We ranked the genes by coefficients after measuring the association between the expression of each gene and the status of the GVITMB under study using logistic regression, controlling for tumor type, age, gender, and calculated patient race. We used these ranked gene lists to perform Gene Set Enrichment Analysis (Subramanian et al., 2005).

Mutational Signature Analysis

We hypothesized that the tumors of some of the patients with GVITMB would exhibit enrichment of mutational signatures. We downloaded all single base substitution signatures from COSMIC (Tate et al., 2019). We determined the optimal contribution of COSMIC signatures to reconstruct the mutational profile observed in each of the patients in TCGA using the R package “MutationalPatterns” (Blokzijl et al., 2018). We converted the contribution values to percentages, such that the sum of the percent contributions of all the COSMIC signatures for each patient was equal to 100%.

We evaluated whether a COSMIC signature is enriched in tumors with a GVITMB by testing for the association between the percent contribution of a signature and the presence or absence of the GVITMB, controlling for tumor type, age, gender, and calculated patient race.

Increased Susceptibility to Mutations in Driver Genes and in the Same Gene Set

To ask if the GVITMB influenced the somatic mutations acquired by the patient, we calculated the number of “probably damaging” somatic mutations in each gene in each patient, as classified by Ellrott et al. (Ellrott et al., 2018). We tested whether the chance of observing a “probably damaging” somatic mutation in the same gene as the GVITMB was more likely in patients with the GVITMB using logistic regression, controlling for tumor type, age, gender, calculated patient race, and clonal nonsynonymous TMB per megabase. We also tested whether the mutational burden in genes of the gene set that the GVITMB was found in differed based on the germline variant status using linear regression, controlling for tumor type, age, gender, calculated patient race, clonal nonsynonymous TMB per megabase and the number of sites for which we were sufficiently powered to call somatic mutations. We controlled for the TMB in both these analyses to test if the mutation burden in these somatically mutated genes was higher than that could be explained by the increase in the overall TMB of the patient.

Validation in an Independent Cohort of Patients with Skin Cutaneous Melanoma Treated with Immune Checkpoint Inhibitors

As part of our analysis, we had identified patients with pathogenic germline variants predictive of TMB in patients with Skin Cutaneous Melanoma (SKCM). We hypothesized that patients with this set of pathogenic germline variants would exhibit a favorable response to immune checkpoint inhibitors (Keenan et al., 2019; Liu et al., 2019; Miao et al., 2018; Van Allen et al., 2015; Van Allen et al., 2014). We, therefore, analyzed sequencing data from 140 patients with skin cutaneous melanoma treated

with immune checkpoint inhibitors (Liu et al., 2019). Although there are a total of 144 patients in this cohort, we only included 140 patients in this study as 4 patients had a “mixed response” to treatment that was not clearly categorized.

We downloaded the raw reads from the non-tumor samples from dbGAP (accession number: phs000452.v3.p1) using the SRA toolkit (http://ncbi.github.io/sra-tools/). The data was aligned and variant called according to GATK best practices (Liu et al., 2019; Van der Auwera et al., 2013). Germline variants were categorized as pathogenic using CharGer (Scott et al., 2019).

We tested whether we were sufficiently powered to detect differences in progression free survival based on the status of the pathogenic germline variants we had identified assuming a hazard ratio of 2 using the “powersurvepi”(https://cran.r-project.org/web/packages/powerSurvEpi/powerSurvEpi.pdf) R package. We were not sufficiently powered to detect associations at the level of individual genes or gene sets. We, therefore, combined all of the gene sets where GVITMB were found in SKCM to create a test gene set for responsiveness to immune checkpoint inhibitors. We were adequately powered to perform the analysis in this larger cohort, as the probability of detecting an association assuming a hazard ratio of 2 was 92.5%. We tested whether GVITMB in this test gene set were associated with progression free survival using Cox regression, controlling for age, gender, treatment type (Nivolumab or Pembrolizumab), prior treatments, and whether or not the patient had brain lymph node, lung, liver, or bone metastases. Cox regression was performed using the “survival” and “survminer” R packages. We tested whether the patients with pathogenic germline variants in the test gene set were associated with increased responsiveness to immune checkpoint

inhibitors based on RECIST criteria using ordinal logistic regression in R. The responses were ordered as follows: progressive disease, stable disease, partial response, and complete response. Our null hypothesis was that the patients with pathogenic variants in genes included in this gene set would not exhibit a favorable response to immune checkpoint inhibitors. To test that hypothesis, we performed one-sided statistical tests when testing for an association between progression free survival and response based on RECIST criteria and germline variants in our test gene set.

Software

Computation was performed using R version 3.5.2. The R packages “ggplot2” and “scatterpie” were used to generate the figures in this manuscript.
